# Mucin1 expression and gustatory function in postmenopausal females: A case-control observational study

**DOI:** 10.4317/jced.59589

**Published:** 2022-11-01

**Authors:** Reham-Lotfy Aggour, Omar Soliman, Olfat Shaker, Sara-Mahmoud Soliman, Amira Abdelwhab

**Affiliations:** 1 Department of Oral Medicine & Periodontology, Faculty of Dentistry, October 6 University, Egypt; 2 Department of Oral Medicine & Periodontology, Faculty of Dentistry, South Valley University, Egypt; 3 Professor of Medical Biochemistry and Molecular Biology, Faculty of Medicine, Cairo University; 4 Department of Pharmaceutics and Industrial Pharmacy, Faculty of Pharmacy, October 6 University, Egypt; 5 Department of Oral Medicine & Periodontology, Faculty of Dentistry, October 6 University, Egypt

## Abstract

**Background:**

Investigating possible relationship between Mucin1 expression levels in saliva, gustatory function, and taste perception in postmenopausal females.

**Material and Methods:**

Using whole mouth taste test, twenty-five post-menopausal females (51.35 ± 5.22 years) and twenty-five premenopausal females (39.65 ± 6.46 years) were prospectively evaluated for gustatory function. The expression of mucin1 was investigated; RNA was isolated from stimulated whole saliva samples and real-time PCR was used to determine mucin1 mRNA levels relative to bactin and GAPDH mRNA levels.

**Results:**

Significant difference was observed between postmenopausal and premenopausal women regarding intensity judgments of all tastants. The difference was more evident for sucrose taste perception (*p*<0.00001). Mucin1 expression levels were significantly decreased in postmenopausal females compared with premenopausal ones (*p*<0.00001). Mucin1 expression level had significant negative correlation with the salt taste sensitivity but did not correlate significantly with intensity judgments of the other tastants.

**Conclusions:**

Postmenopausal women have a reduced gustatory function, especially sucrose. Mucin1 expression was significantly decreased in postmenopausal females and had a significant negative correlation with the salt taste sensitivity. However, no correlation was found between mucin1 expression level and taste sensitivity of other tastants.

** Key words:**Gustatory function, MUC1, taste, postmenopausal.

## Introduction

Oral homeostasis relies on proteins and mucins present in saliva and coating all oral surfaces. Mucins are mainly divided into secreted-gel-forming mucins and membrane-bound mucins (1). The roles of membrane-bound mucin molecules, such as Mucin1 (MUC1) and its shed soluble form, in the oral cavity have not been sufficiently explored. MUC1, a large transmembrane glycoprotein, is an epithelial surface bound molecule expressed on the apical surfaces of most secretory epithelia including the salivary glands and the oral epithelium (2).

MUC1 present on epithelial cell surfaces after secretion has extended negatively charged carbohydrate chains and plays a central role in epithelial defense by increasing the salivary proteins anchoring forming network structures with secretory mucins and other proteins. Moreover, as a transmembrane mucin, MUC1 acts as sensor of environmental changes in the epithelium (3). The change in MUC1 expression may trigger changes in signaling to the oral mucosal epithelial cells (4). Cleavage or dissociation of MUC1 molecules by diverse ligand binding to the extracellular domain can transfer information to the cytoplasmic tail, thereby initiating intracellular signaling, contributing to the ability of cells to respond to the external environment (5).

Except for studies about altered expression of MUC1 in squamous cell carcinoma and oral potentially malignant disorders (6), studies on the association of oral epithelial MUC1 with oral diseases or conditions are sparce. Pramanik *et al*. (7) related the decreased oral lubrication in dry mouth patients with a lower expression of MUC1 (7). Kho *et al*. (8) reported increased MUC1 expression in the oral mucosa of patients with burning mouth syndrome (BMS) compared with patients with oral lichen planus or normal controls (8).

Taste is defined as the ability of the individual to respond to dissolved molecules in saliva “tastants”. Taste plays a pivotal role in food preferences and dietary habits and consequently the nutritional status, quality of life and general health of individuals. Alteration of gustatory function is a common condition, and dentists may be the ﬁrst clinicians who can establish taste dysfunction (9). The etiology of taste disturbances is multifactorial and there are numerous local and systemic disorders which can affect taste perception. Additionally, many drugs do cause a major impact on taste sensation. Several physiologic conditions may result in taste alterations, One of these conditions is menopause (10).

Postmenopausal changes occur in numerous organs including taste buds. Some studies have investigated the possible causes of taste dysfunction in postmenopausal females and controversial results are reported (11,12). Saliva constantly bathes the taste buds and tastants can access taste receptors by diffusion throughout the salivary mucosal pellicle. Altered salivary rheology may predispose to gustatory dysfunction by affecting oral mucosal defense mechanisms and sensitivity to oral stimulants (13). The transmembrane oral MUC1 may facilitate muco-adhesion of the salivary pellicle by binding to other salivary mucins (4). This binding protects the oral mucosal surfaces, bathing the taste buds, and providing a trophic stimulus (14).

No previous studies have evaluated relationship between MUC1 expression and gustatory function in postmenopausal females. Given the potential that MUC1 is involved in maintaining gustatory function, additional studies seem prudent. Accordingly, the present study was performed with the following objectives.

• Evaluating the probable alterations of the gustatory function in postmenopausal females 

• Investigating possible association between MUC1 expression in saliva and gustatory function in postmenopausal women.

We hypothesized that postmenopausal females have less gustatory function than premenopausal females and this alteration may be related to altered MUC1 expression level. This study may shed light on role of MUC1in oral health.

## Material and Methods

This observational case– control study included fifty patients, who were recruited from patients attending Oral Medicine and Periodontology department, Faculty of Dentistry, October 6 university, between 1 November 2020 and 30 January 2021. The research protocol was reviewed in compliance with the Helsinki Declaration and approved by the institutional ethics committee (#RECO6U/4-2020) and informed consents were obtained from all participants after explaining the research study in detail. The study conforms to the reporting requirements of the STROBE guidelines. Power analysis was performed to calculate minimum number of patients for the study. To achieve a significance level (type 1 error) as 0.05 and power (type 2 error) as 0.8, it was decided to include 25 subjects for each group. Individuals were divided into.

i. Study group which included postmenopausal females, at least two years after beginning of the menopause.

ii. Control group which included premenopausal females recruited randomly among volunteer patients.

Inclusion criteria for the study group were as follows: postmenopausal females who 1) age below 60 years old, 2) with complete amenorrhea at least two years after beginning of the menopause, 3) are not taking any medication that affect taste perception or salivary flow rate [including hormone replacement therapy (HRT)], 4) are not a denture wearer, 5) having good oral hygiene, 6) had no oral mucosal lesions and 7) had unstimulated salivary flow rates > 0.1 mL/min. Inclusion criteria for the control group are similar to the study group except that individuals are healthy volunteers in the age range between 30-50 years with regular menstrual cycles and not pregnant or breast-feeding women. Exclusion criteria for all participants were 1) smokers, 2) patients having any systemic conditions including endocrinological, respiratory, neurological, psychological and nutritional diseases and 3) those who have a history of radiotherapy and/or chemotherapy in the head and neck and 4) subjects with problems in communication.

a. Evaluation of gustatory function

For all participants, clinical evaluation procedures included dental, oral, and periodontal examination and measuring salivary flow rate. A questionnaire was utilized to assess subjective symptoms (mucosal burning, oral dryness, parathesia, gingival bleeding, mucosal lesions, pain and bad breath). Participants were asked to fill in a scale (ranging from 0 to 100; 0 = My taste function is very poor, 100 = I have extraordinarily good taste function). Blood tests were carried out to rule out possible systemic factors that may cause altered taste sensations. The tests included complete blood picture, ferritin, zinc, vitamin B12 and folate. Blood glucose, liver function tests and renal function tests were also evaluated.

For testing gustatory function, a whole mouth above threshold taste test was performed in which a concentration of sucrose, sodium chloride, citric acid and quinine hydrochloride solutions were used for sweet, salty, sour, and bitter tastants respectively. Quality judgments and intensity ratings for each tastant solution was assessed. Five concentration levels (in ½ log steps) of sodium chloride (0.01–1.0 mM), citric acid (0.032–0.32 mol l-1), quinine hydrochloride (0.01– 1.0 mM) and sucrose (0.01–1.0 mM) were prepared in samples of 5 ml each using distilled water. For each solution, the intensity of taste perception was determined by setting the lowest concentration as 1’ and the highest concentration as 5’. Each tastant presented to the patient in a cup in addition to two other cups of distilled water on a tray in a random order. Subjects were instructed to rinse with water, then hold the test solution in the mouth for 15 seconds and then expectorate the solution thereafter. The subjects were asked to choose one of four tastes to describe the administered taste. The solutions were given in rising concentrations until the individual tastant was detected. The lowest concentration at which the subject perceived the taste was defined as the detection threshold. The taste score was determined based on the detection threshold. According to the concentration corresponding to the identified taste, the subject received a score between 0 and 5 for each administration. If subjects did not recognise the taste at the No. 5 dilution concentration, they received a score of 0; that is, the lower the threshold, the higher the assigned score (15). Quality assessment was coded as correct, incorrect or tasteless by asking participants to name the taste they recognised (16). All measurements were documented in the morning between 10:00 am and 12:00 am, at least two hours after abstaining from eating and drinking. The solutions were weekly prepared. The whole evaluation procedure was completed within 15 minutes.

b. Analysis of oral mucosal MUC1 expression level:

Saliva samples were collected in the morning between 10:00 am and 12:00 am. Patients were instructed to abstain from eating and drinking at least 2 hours before sample collection. To stimulate salivary flow, patients are given 1 g of gum base to chew. After discarding the saliva that was collected for the first 2 min, the stimulated whole saliva was collected for the next 10 minutes. Immediately after collection, samples were stored on ice, aliquoted to minimize freeze thaw cycles, and stored at −80°C for RNA extraction.

Determination of oral mucosal epithelial MUC1 expression level 

Five ml saliva were centrifuged at 2500 g for 10 min at 4°C. The supernatant was then collected and centrifuged at10,000 g for one min. The resultant supernatant was transferred into a new tube, and RNA extraction was processed immediately. RNA in supernatant (200 µl) was isolated using the miRNeasy extraction kit (Qiagen) according to the manufacturer’s protocol. Extracted RNA was quantitated using NanoDrop_ (ND)-1000 spectrophotometer (NanoDrop Technologies, Inc.). The extracted RNA was reverse transcribed by using QuantiTect Reverse Transcription (RT) Kit (cat No 205310) Qiagena, Germany. The RT mix was incubated for 15 min at 42°C followed by 3 min at 95°C to inactivate the reverse transcriptase enzyme. Quantitative real-time PCR was performed using specific primer Hs_MUC1_1SG QuantiTect primer assay (QT00015379) provided by QuantiTect. Sybr green PCR kit Qiagene (cat no. 208052) was used for amplification. Glyceraldehyde 3-phosphate dehydrogenase (GAPDH) was used to normalize the expression pattern and for relative quantification of the genes using the ΔCt method. The real-time cycler was used for 40 cycles then melting curves analyses were performed. The cycle threshold (Ct) value is the number of qPCR cycles required for the fluorescent signal to cross a designated threshold. ΔCt was calculated by subtracting the Ct values of GAPDH from those of target mRNAs. ΔΔCt was calculated by subtracting the ΔCt of the control samples from the ΔCt of the test samples. The fold change of MUC1 expression was calculated by the equation 2–ΔΔCt.

-Statistical analysis 

Statistical analysis was performed by the MedCalc statistical software version 18.10.2 (MedCalc Software, Ostend, Belgium). The Mann-Whitney U-test was used to calculate differences between the groups. The Spearman’s correlation analysis was used to determine the potential correlation between Muc1 expression and subjective/objective taste perception. A *p-value* less than 0.05 was considered statistically significant. While the sample collection was performed by the same investigator, a code was assigned to each sample and only broken after completing the analyses.

## Results

Fifty subjects were included in the study. For the study group, 25 postmenopausal females (51.35 ± 5.22 years) were recruited. Twenty-five premenopausal females (39.65 ± 6.46 years) represent the control group. The questionnaire revealed that 8 (32%) postmenopausal patients complained of taste alteration. The mean (±SD) severity of altered gustatory perception was 40.7 ± 10.3. Eight patients (32%) complained of burning sensation. No other symptoms have been reported

Regarding correct quality identification, both groups detected all tastants correctly and the intergroup differences were statistically non-significant (*P* > 0.05). For all tastants, measured taste function was significantly lower in postmenopausal women compared to controls (*P* < 0.05) ( Table 1, Table 2). For sucrose, taste perception intensity was significantly lower in postmenopausal females than taste perception intensity of other tastes (*P* < 0.00001) as shown in Table 1. The taste scores of the four tastes were summated to obtain an overall evaluation of gustatory function (Table 2).

**Table 1:**
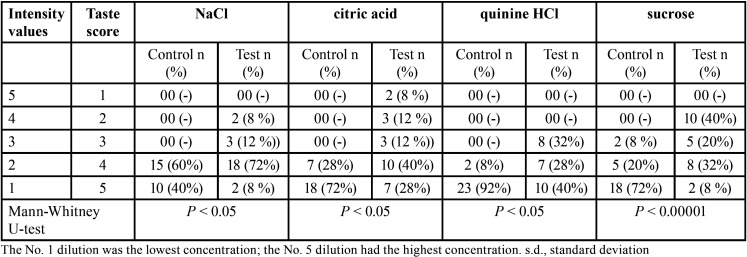
Taste intensity for NaCl, citric acid, quinine HCl and sucrose solutions in test and control groups.

**Table 2:**
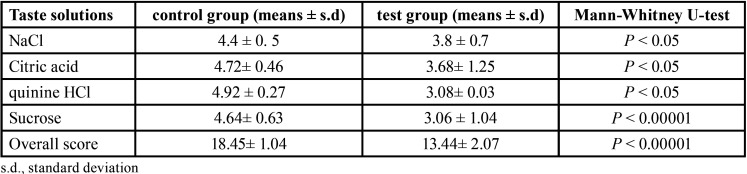
Mean total taste scores in test and control groups.

-Expression levels of MUC1 

Significant decrease was detected in MUC1 transcripts in the test group compared with the control group (*p* < 0.00001) (Table 3). In the test group, MUC1 expression shows significant negative correlation with intensity of salt perception (r=-0.876, *p*<0.001). No correlation was found between expression level of MUC1 and taste sensitivity of other tastants (*P*> 0.05). Similarly, no correlation was found between MUC1 expression level and Subjective taste perception (*P* > 0.05) (Table 4).

**Table 3:**
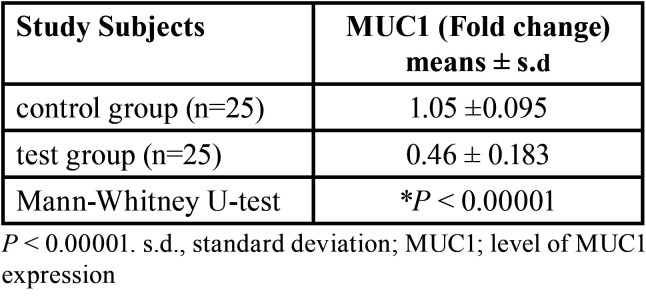
MUC1 in stimulated whole saliva from control and test groups.

**Table 4:**
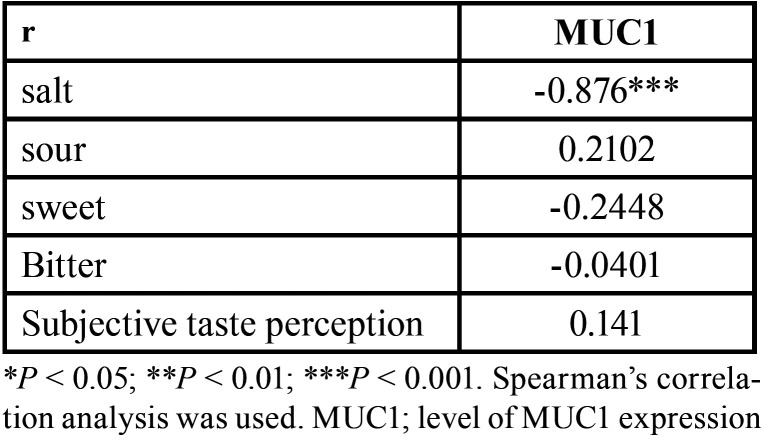
Correlation coefficients (r) between MUC1 expression level and gustatory function (taste scores) and subjective taste perception in test group.

## Discussion

The principal finding in the current study is the significantly reduced intensity of taste perception for all tastants in the postmenopausal women. Additionally salivary MUC1 expression is significantly decreased in postmenopausal women compared with premenopausal ones. Interestingly, MUC1 expression level had significant negative correlations with the salt taste sensitivity but did not correlate significantly with intensity judgments of the other tastants. It is the first clinical study exploring possible correlation between altered gustatory function and salivary MUC1 expression level.

To decrease the effect of confounding factors, we excluded patients with any oral or dental pathosis especially periodontal diseases. Upregulation of MUC1 expression has been observed after treatment with pro-inflammatory cytokines or *Porphyromonas gingivalis* (17). Additionally, we used stimulated saliva samples in the current study since some studies failed to detect MUC1 in unstimulated whole saliva (7).

In accordance with previous studies (18,19), the significantly reduced intensity of taste perception reported in the current study is more pronounced regarding sucrose tastant, reflecting a decline in sweet sensitivity. Unlike the current study, a recent Japanese study found no significant difference in taste perception between premenopausal and postmenopausal females (20). Perhaps, the different results are attributed to ethnic differences or contribution of other chemosensory functional factors.

The decrease in salivary MUC1 level in the current study could be explained by proteolysis of its peptide core and/or decreased secretion of mucin associated with the psychoendocrinological changes reported in postmenopausal women. A positive correlation between level of progesterone and oral mucosal MUC1 is reported in previous study, however, the authors claimed that hormonal changes associated with psychological stress might also affect oral mucosal MUC1 expression (21). Limited studies have investigated the effect of gonadal and stress hormones on oral mucosal MUC1. Lee *et al*. (22) reported that oral mucosal MUC1 expression was negatively correlated with salivary cortisol/DHEA through the entire menstrual cycle and negatively correlated with progesterone, principally, during the mid-luteal phase. The authors concluded that stress-related endocrinological interplay decrease MUC1 expression in healthy young females (22). Chang *et al*. (23) reported decreased oral mucosal epithelial MUC1 expression in older adults compared to young adults (23). However, Kang *et al*. (21) reported higher MUC1 expression in post-menopausal BMS patients than matched controls (21). A compensatory response to repeated irritation has been suggested as a possible mechanism of this increase. The latter is the only study that has focused on MUC1 expression in postmenopausal women with symptomatic oral manifestations, however, they study didn’t explore the relation between MUC1 level and gustatory function.

In the present study, the taste perception and gustatory function did not correlate with MUC1 expression except for salt tastant. Interestingly, a significant negative correlation between MUC1 expression and salt tastant sensitivity was found. However, to made conclusions regarding relationship between MUC1 expression and gustatory function, further studies with greater numbers of subjects are required. Based on accessible literature, causes of taste dysfunction in postmenopausal females are multiple and involve the interplay between biological and psychological systems. Gustatory perception is very complex and under the influence of many factors. Investigating the functionality of MUC1 which is affected by levels of sialylation and glycosylation and effects on binding capacity could add significant information. In their valuable study, Pushpass *et al*. (24) demonstrated a reduction in the quantity and glycosylation of salivary mucins in older adults (24). The authors suggested that altered muco-adhesiveness of saliva may be essential for gustatory function as the adhered mucins yield an extracellular matrix that aids concentration of tastants near taste receptors. However, they claimed that this mechanism seems unlikely for more hydrophilic tastants like sugar and salt (24). Unveiling MUC1-dependent pathways important for signal transduction may aid in clarification of its role in gustatory function taking in consideration that mechanisms may differ for each tastant.

In the current study, we compared premenopausal to postmenopausal women because regarding gender, some studies (25,26) reported that women mostly demonstrate a more sensitive sense of taste than males and men are more liable to losses than women. Females that became menopause at least 2 years before participation are included because it has been reported that levels of gonadal hormonal have reached stabilization about two years after menopause (27). To minimize the effect of age on gustatory function, age below 60 years old was selected (22).

Taste perception is a psychophysical process. Subjective symptoms reported by patients and objective outcomes by testing gustatory function should be related to each other. In the present study, a whole mouth taste test was used instead of regional taste-testing systems which have some limitations. Regional taste testing system may result in poor diffusion and exact control of the extent of the stimulus on mucosal surfaces may not be achieved. However, whole mouth test of gustatory function (used in the current study) is suitable for assessing everyday’ taste experiences that are not mirrored by regional tests (26). The fact that every taste bud has some degree of sensitivity to all of the main taste sensations also supports the use of the whole mouth taste test.

In this study, taste impairment was reported by only 8 (32%) of postmenopausal females. In the remaining, the decreased gustatory function was totally unnoticed. Previous studies reported low specificity and high error rate of subjective taste evaluation using questionnaires and showed that subjective self-reported taste alterations do not necessarily reflect measured objective gustatory function (15,16). This further emphasizes that the only way of drawing an accurate picture of taste function is measuring it using standardized methods.

In the current study, despite the significant difference between postmenopausal and premenopausal women regarding intensity judgments of all tastants and the decreased salivary MUC1 levels in postmenopausal females when compared with premenopausal ones, we can’t attribute decreased gustatory function in postmenopausal women to altered salivary mucosal epithelial MUC1 expression. The findings should be interpreted with caution due to limited number of patients included in the present study. Other factors should be evaluated such as properties of saliva and salivary mucins functionality. Studies with larger sample size are recommended. Moreover, comparison between postmenopausal females complaining of dysgeusia and other who have no subjective taste alterations is recommended.
